# 

**Published:** 1870-04

**Authors:** 


VOL. XXIV. No. 4.

### THE
### Dental Register.

EDITED BY

J. TAFT & G. WATT.

APRIL, 1870.

MONTHLY:

THREE DOLLARS PER ANNUM, IN ADVANCE.

CINCINNATI:
WRIGHTSON & CO., Printers, 167 WALNUT STREET.

*J. & WM. TAFT, Dentists, 117 West Fourth St.*

